# Typhoid Infection

**Published:** 1877-12

**Authors:** 


### TYPHOID INFECTION.

We are indebted to one of our recent exchanges for the following abstract of experiments, performed by M. J. Guerin. He wished to determine whether the stools in typhoid fever had a poisonous action. He sought more especially to determine whether the dejections of typhoid fever patients contained an infecting matter from the beginning:—

1. Subcutaneous injections of diarrhœic typhoid stools were made on twelve rabbits. Ten of the rabbits died in the course of the first four days, while one died a month after, and one recovered.

2. The heart's blood of a rabbit which had died on the third day after the injection of the typhoid stool was inoculated in another rabbit. This rabbit died on the next day. Injections of fæces which came from individuals suffering from other diseases were without any results.

3. Fæces, intestinal blood, urine, detritus of swollen mesenteric glands, and constituents of intestinal ulcers which came from a typhoid fever patient were inoculated on twelve rabbits. These all died at the latest thirty hours afterwards, with severe general symptoms. Three of them had diarrhœa Post mortem examination showed no characteristic changes.

4. Material which came from typhoid fever patients (blood, urine, and fæces), and which had been kept four months, was inoculated on six rabbits. All these animals died without showing characteristic changes on dissection.

The conclusions which the author arrives at from his experiments are as follows:—

1. Typhoid stools immediately after being passed contain a poison which kills rabbits in a short time.

2. The blood and urine of typhoid fever patients has the same quality, as does also the detritus of swollen mesenteric glands, and typhoid intestinal ulcers.

3. This property is not lost by keeping the material for months.

4. The fæces of healthy individuals or of those suffering from other diseases do not possess this property.—*Boston Med. and Surg. Journal.*

